# A protocol for an individualised, facilitated and sustainable approach to implementing current evidence in preventing falls in residential aged care facilities

**DOI:** 10.1186/1471-2318-10-8

**Published:** 2010-02-17

**Authors:** Betty Haralambous, Terry P Haines, Keith Hill, Kirsten Moore, Jennifer Nitz, Andrew Robinson

**Affiliations:** 1 Preventive and Public Health Division, National Ageing Research Institute, 34-54 Poplar Rd, Parkville, 3052, Australia; 2 Allied Health Clinical Research Unit, Southern Health, Kingston Centre, Kingston Rd, Cheltenham, 3192, Australia; 3 Physiotherapy Department, Monash University, McMahons Rd, Frankston, 3199, Australia; 4 Physiotherapy Department, University of Queensland, Therapies Lane, St Lucia, 4072, Australia; 5 Musculoskeletal Research Centre, LaTrobe University/Northern Health, Bundoora, 3086, Australia; 6 School of Health Sciences, The University of Melbourne, Queensberry Street, Carlton 3010, Australia; 7 Division of Physiotherapy, The University of Queensland, Sir Fred Schonell Drive, Brisbane, 4072, Australia; 8 School of Nursing and Midwifery, University of Tasmania, Private Bag 121, Hobart, Tasmania 7001, Australia

## Abstract

**Background:**

Falls are common adverse events in residential care facilities. Commonly reported figures indicate that at least 50% of residents fall in a 12 month period, and that this figure is substantially higher for residents with dementia. This paper reports the protocol of a project which aims to implement evidence based falls prevention strategies in nine residential aged care facilities (RACFs) in Australia. The facilities in the study include high and low care, small and large facilities, metropolitan and regional, facilities with a specific cultural focus, and target groups recognised as being more challenging to successful implementation of falls prevention practice (e.g. residents with dementia).

**Methods:**

The project will be conducted from November 2007-November 2009. The project will involve baseline scoping of existing falls rates and falls prevention activities in each facility, an action research process, interactive falls prevention training, individual falls risk assessments, provision of equipment and modifications, organisation based steering committees, and an economic evaluation. In each RACF, staff will be invited to join an action research group that will lead the process of developing and implementing interventions designed to facilitate an evidence based approach to falls management in their facility. In all RACFs a pre/post design will be adopted with a range of standardised measures utilised to determine the impact of the interventions.

**Discussion:**

The care gap in residential aged care that will be addressed through this project relates to the challenges in implementing best practice falls prevention actions despite the availability of best practice guidelines. There are numerous factors that may limit the uptake of best practice falls prevention guidelines in residential aged care facilities. A multi-factorial individualised (to the specific requirements of each facility) approach will be used to develop and implement an action plan in each participating facility based on the best available evidence.

## Background

Falls are common adverse events in residential aged care facilities (RACFs). Reported figures indicate that at least 50% of residents fall in a 12 month period, and that this figure is substantially higher in some at risk populations such as people with dementia where the incidence is 70-80% [1]. Injuries from falls are also more common for RACF residents than for older people living in their own home, with a study by Kallin et al (2002) reporting injuries associated with 54% of falls, and a third of injuries being fractures [2]. Another study identified that over half of the fractures sustained from falls by residents were hip fractures, which have an enormous associated personal and societal cost [3]. Although accurate cost data is not available for costs associated with falls in RACFs in Australia, some data from Europe estimates the costs associated with falls in residential care to be 994EURO/fall (approximately $AUD1,560) [4].

There are clear benefits of a multiple strategy approach to falls reduction for older people in residential care settings, both as an intervention and prevention strategy [5]. Randomised trials have shown that multifactorial interventions, either based on falls risk assessments, or a range of general interventions introduced for all residents can reduce falls in residential care settings [6-8]. Single interventions including medication review [9], vitamin D supplementation [10,11], continence management combined with functional training [12], and staff education [13] have also been shown to reduce falls. Other interventions such as exercise have had mixed outcomes in residential care settings [14-16]. Approaches such as providing information for residents and carers, and strategies to improve observation or surveillance of at risk residents (e.g. bed and chair alarms) are recommended as part of best practice in residential care settings, although there are no randomised trials evaluating the effectiveness of these approaches [5].

Previous reviews have shown that the involvement of staff, the method of training provision, staff awareness of falls and staff implementation of evidence based strategies to prevent falls and falls related injuries are areas requiring further exploration [5,17].

Despite the availability of evidence-based guidelines for implementing falls prevention and other interventions in residential aged care, there is moderate evidence that these interventions are not routinely or easily incorporated into routine practice. For example, in a study aiming to implement pain management guidelines and falls prevention guidelines in residential care settings, less than 45% of facilities participating in a training program regarding the guidelines actually implemented the one or the other of the guidelines [18]. While there was some evidence of practice change in line with the guidelines in some of the facilities, a range of barriers to implementation were identified.

Action research is an approach that could be useful in engaging staff in falls prevention interventions and for achieving sustainable practice change. Action research is a process where staff are supported to undertake an investigation into their current practice, recognise problems, envisage possible solutions and take action with the intentions of working towards addressing the problem. Utilising this approach, groups of staff collaborate to take action to identify context specific issues, identify and implement an evidence based approach and in the process support and challenge each other to make progress on the problem [19]. Action research is a method of choice when a group of people have a desire to work collaboratively to develop new understandings of their situation to address practical problems through locally specific and contextually appropriate forms of action [20-22].

In addition to limitations implementing evidence based practice, few studies have investigated the financial implications of falls prevention strategies in the residential care setting [5].

The primary aim of the project is to implement evidence based falls prevention guidelines in nine RACFs in Australia with the intent to reduce falls and falls-related injuries. The approach to be adopted involves multiple strategies to improve falls management. These include: improving RACF staff knowledge and attitudes with respect to falls risk; actively engaging a core group of staff in a process of designing, implementing and evaluating local specific interventions to reduce falls in the context of their participation in action research falls groups in each RACF, and; identifying a range of falls risk factors for individual residents (e.g. undertaking falls risk assessments) and for facilities (e.g. environmental falls risk factors). This project also encompasses an economic evaluation for which additional data to support economic variables will be collected.

The project is funded by the Australian Government Department of Health and Ageing, as part of the "*Encouraging Best Practice in Residential Aged Care*" (EBPRAC) program. The EBPRAC program aims to improve evidence-based clinical care for residents in aged care homes and to enable nationally consistent application of this care.

## Method

The project will involve baseline scoping of existing falls rates and falls prevention activities in each RACF, an action research process, interactive falls prevention training, individual falls risk assessments, provision of equipment and modifications, organisation based steering committees, and an economic evaluation. In all RACFs a pre/post design will be adopted with a range of standardised measures utilised to determine the impact of the interventions. The study involves fostering ownership by each facility of an evidence based, best practice falls prevention approach through involvement of staff in partnership with experts in falls prevention using best practice resources to guide the process.

### Recruitment of facilities and participants

RACFs were approached in three states across Australia, and all accepted the invitation to participate in the project. These facilities were selected purposively to represent a range of sizes, target groups, and geographic features, including high and low level care, dementia specific, rural/regional, metropolitan, ethnic specific or psychogeriatric. All except one facility are not for profit organisations and some are linked to health services. Across the nine participating facilities approximately 670 people reside and 650 staff are employed. All staff and residents will be invited to take part in at least one component of the project.

Each facility is allocated funding to release a nurse staff member to the role of key contact and project support person known as the falls resource nurse (FRN, 0.1 EFT) for the project. The secondment provides an opportunity for the staff member to undertake project activities without being distracted from their existing workload. It is anticipated that this process would contribute to ongoing sustainability through building capacity within the facility. An additional 0.1 EFT funding is provided to support other areas of staff time and engagement in specific falls prevention activities.

With the assistance and advice of the FRN in each of the facilities, residents and families will be informed of the research. Methods of informing and engaging residents and families will include attendance at family meetings, flyers distributed on family and resident notice boards and distribution of project information through facility mail outs.

### Procedure

#### Baseline scoping

To gauge how well facilities are currently implementing evidence based practice, a comprehensive scoping audit will be undertaken at the beginning of the study. This will include: falls incident data, an audit of the current falls prevention activities, environmental audits, a survey on resident safety culture within the organisation and a questionnaire on sustainability.

From each facility, falls data for the six months preceding project commencement will be analysed, including data on circumstances, precipitating factors, consequences and management. These will be reported as falls/1000 resident bed days. Frequency analyses will be used to determine the most common locations, circumstances and consequences of falls.

The falls scoping audit was developed using key falls prevention strategies identified in the Victorian Quality Council's falls prevention guidelines [23] and covers issues such as falls risk assessment, collection and use of falls prevention data, and access to allied health staff. Key interventions covered in the audit include: group exercise, individual exercise, walking aids, hip protectors, medication review, vitamin D and calcium supplementation, toileting assistance program, adequate condition and use of aids for sensory loss, feet in good condition and appropriate footwear, surveillance, hi-lo or lo-lo beds and referral to other health professionals. The Project Officer will send the survey to the FRN to complete. After the FRN completes the survey, the Project Officer will go through it with the FRN to make sure everything is completed and that the Project Officer has full knowledge of the falls prevention activities taking place in the facility.

To identify environmental hazards that may require modification, environmental audits will be completed in each facility. This will involve an audit of resident rooms, indoor and outdoor communal areas of each facility using an environmental audit tool adapted from a tool developed to improve the environment for older people in health services [24]. Recommendations from the environmental audits will be made to participating facilities, and used to assist in the allocation of funds available for environmental modifications as part of the project.

A safety culture survey for residential aged care facilities will be used to assess safety culture and hazard reporting at baseline to inform action research. This survey has been adapted from the safety culture survey for hospitals [25]. The survey will be researcher administered to all facility staff working within a 24 hour period, including staff working on night, day and evening shifts.

To measure how each facility will sustain project activities, the sustainability model questionnaire will be completed with the action research groups [26].

At the completion of the scoping phase, an individualised report will be provided to each facility, highlighting areas being well implemented, and areas where additional activity needs to be targeted. This will form the basis for the Action Research Groups to identify gaps in evidence based practice to address in the implementation phase within each facility.

#### Action research

An action research approach will be used in the facilities as a key element to work towards achieving the project aims. In each RACF staff interested in falls prevention will be invited to join a falls action research group (FARG). To recruit staff, meetings will be held in each RACF to identify volunteers. Eligibility for FARG membership will include any staff member working within the facility (for example, nurse, personal carer, lifestyle, allied health, kitchen, laundry, cleaning staff, management). In some instances it might be relevant to include staff who may not work in the facility but visit on a clinical basis such as a physiotherapist. Five-six staff members will be recruited in each FARG. A project officer will facilitate meetings that will be audio taped and transcribed. The project officer will conduct a first level analysis that will be distributed to the respective FARG members prior to the next meeting to ensure the discussion has been recorded accurately and key points included.

From May 2008 FARG members will meet fortnightly for 12 months to identify key issues, facilitators and barriers to successful falls prevention within their RACF. During these discussions the members will articulate their position on the issues that impact on the occurrence of falls in their facility. Each FARG will draw on findings together with the audit data to determine site specific activities on which to focus their interventions to improve falls prevention in the RACFs.

These activities will be developed into action plans that the FARGs will then implement with support and advice from the project officer. Methods for evaluating these actions will be established and monitored by the FARGs. Project officers will assist in writing up action plans incorporating evaluation measures.

The project officers facilitating the FARGs in each RACF will also maintain a qualitative record of the group's activities or project diary that will provide useful data for determining factors that enabled or prevented the success of the falls interventions. This will be supplemented with thematic analysis of transcripts using NVivo software.

To measure FARG participants' perception of professional clinical practice (such as communication, leadership, teamwork, control) the Revised Professional Practice Environment Scale (RPPE) [27] will be used. It will be distributed and collected by project officers at the first and last FARG meetings for all action research staff to complete.

#### Training and support

Prior to commencing the FRNs will be provided with a one day training program on falls prevention, action research, practice change and other aspects of the project, such as data collection processes. A test of knowledge of falls risk factors and prevention interventions will be completed by FRNs at the beginning of the training session. One week after the training program, FRNs will be emailed the test and asked to fill it in and return it without referring to any written materials and allowing approximately an hour to complete.

In addition to the training program, FRNs will be provided with a resource pack which will incorporate key information from international best practice guidelines such as the RNAO guidelines (Canada) [28] and the NICE guidelines (UK) [29] as well as the Victorian Quality Council falls prevention guidelines [23], the Australian Safety and Quality Council's guidelines [30]. FRNs and FARG members will also have access to expert advice from the multidisciplinary research team including physiotherapists, nurses, social workers and a geriatrician. The FARG will also have an initial training session with a nurse experienced in undertaking action research in these settings. Project officers will also have regular teleconferences with this nurse to discuss action research processes and discuss any problems arising.

Facility wide interactive falls prevention training will be run at each facility. Development and implementation of the program will be tailored to the specific needs of each facility and may be part of the action plan developed in the FARGs. The training will aim to spread the message that falls prevention is everyone's responsibility.

#### Falls risk assessment

The Falls Risk for Older People - Residential Care (FROP-RC) will be completed for all consenting residents [31]. This tool will be modified slightly from a previously validated falls risk assessment tool for the sub-acute hospital setting [32]. The tool will either be completed by the FRNs, other FARG members and/or with an external allied health professional depending on the capacity of each facility to conduct the assessments as well as the decisions of the FARGs. The individual resident falls risk assessments will be used to inform individualised strategies. The assessments will also identify falls risk issues that are able to be addressed facility wide. Data will be entered into an excel spreadsheet for analysis. The research team will provide facilities with trend data, e.g. a high proportion of residents having a particular modifiable risk factor.

#### Equipment and modifications

The project budget allows a set allocation of funding for each facility to spend on environmental modifications, hip protectors for residents with high risk of repeated falls/injury and one or two hi-lo or lo-lo beds. The use of these funds is at the discretion of FARGs and managers in each facility, with approval from the external project manager. The environmental audit undertaken in the scoping phase will include a list of areas that could be improved in each facility. Facility staff will use this list to determine which modifications they think will be most useful in preventing falls in their facility.

#### Economic evaluation

A cost effectiveness analysis will be conducted as a part of this project from societal and health-service provider (residential aged care facility) perspectives. The costs of program implementation will be tracked during project implementation and valued at market rates where available. Costs averted from the prevention of falls and fall-related injury will be calculated by comparing fall and fall-injury rates before and after intervention commencement with modelling of cost-per fall data captured from two of the participating sites. This cost-per falls data will include costs of hospitalisation to treat fall-related injuries, costs of transportation to hospitals, and costs of visits by and to general practitioners/other health professionals to treat fall-related injuries.

#### Steering committees

Within each facility an organisation based steering committee will be established. There are two instances where two facilities are part of one organisation and are indicating a preference for a joint steering committee to be formed, resulting in a total of seven committees. The purpose of each committee is for the research team to consult with key players within the organisation throughout the duration of the project and to keep them informed of project progress and outcomes. Key responsibilities of the committee will include: overseeing the project at an organisation level; monitoring research activities and providing feedback regarding research findings; being a resource to the research team and providing advice from an organisation specific perspective; and discussing strategies for maximising sustainability after project completion.

#### Evaluation

Falls incident data will be reviewed by the project team on a six monthly basis throughout the project and will be reported back to facilities at six and 18 months. As previously described, each FARG will also develop and complete site-specific evaluations relevant to the action plan implemented. After completion of the FARGs, the sustainability model questionnaire, the falls scoping survey and the safety culture survey will also be readministered to gauge any other changes during the project.

Final resources for dissemination of project processes and outcomes will be developed and disseminated. Findings will be collated into facility specific reports for facilities to review outcomes as well as an overall final evaluation report combining data from all sites and incorporating the economic evaluation.

### Analysis

Data from the questionnaires and surveys will be analysed using the appropriate non-parametric statistical analyses. Responses to individual items of the survey of safety culture will be compared between groups using ordinal logistic regression analysis, clustering results by the site from which the data was collected.

The rate of falls per resident observed year will be compared between pre intervention and during intervention period using a generalised estimating equation [33]. The monthly number of falls at each facility is the individual unit of measurement. Monthly data by facility will be coded by whether it is collected during the pre-intervention or intervention period. Intervention period data will be additionally coded by whether the data is collected during an "active" intervention period or during an "inactive" intervention period (after completion of the FARGs). Generalised Estimating Equations will be conducted using a Poisson outcome measure distribution family and an exchangeable working correlation structure, though the correlation structure will be checked prior to analysis to determine if the exchangeable structure is still the most appropriate. Adjustment will be made for the number of resident observed years at each site per month. Pre/during intervention and active/inactive intervention variables will be entered into this model as explanatory variables.

For the economic evaluation, the change in costs allocated for the prevention of falls and treatment of fall-related injuries between the pre-intervention and during intervention periods will be the numerator. The change in the number of falls per month (adjusted for resident observed years) will be the denominator, culminating in a cost per fall prevented ratio. Several cost-effectiveness models will be developed by modelling an extended time-line of the project duration for +1, +2, +3, +4 and +5 years beyond the cessation of the implementation phase. The difference between active and inactive period fall rates will be used to guide this modelling. Further sensitivity analyses will be conducted by modelling clinically reasonable variations in the key outcome (falls) and cost (e.g. staffing) measures collected.

#### Power analysis

Data from a previous falls prevention trial in the residential aged care setting indicated that falls per resident year (broken into two-month blocks) captured over a 16 month period had a mean (sd) of 2.6 (0.7) [34]. Assuming a similar rate and standard deviation of falls in our present study, our trial across nine facilities will have 85% power to detect an absolute reduction in the rate of falls of 0.7 (this would be a 27% reduction in the rate of falls relative to a baseline of 2.6 falls per resident year).

### Ethics

This project was approved by local human research ethics committees for each site (Ballarat Health Service & St John of God Human Research Ethics Committee; Toowoomba & Darling Downs Health Service District Human Research Ethics Committee; Human Research Ethics Committee St Vincent's Hospital; Tasmania Social Sciences Human Research Ethics Committee).

All staff in each facility will be provided with a Plain Language Statement and Consent Form inviting their participation in the study (for example, training, focus groups, FARGs, surveys, reviews of various documents and tools).

Managers and/or FRNs will identify residents who will be able to give informed consent and provide them with a Plain Language Statement and Consent Form to obtain consent for various components of the project that are outside usual care, such as audits to their rooms, additional falls risk assessment, or implementation of safety devices such as hip protectors. For those residents unable to provide informed consent due to cognitive impairment, facility staff will provide relatives/contact persons with a Plain Language Statement and Consent Form to seek consent for participation on behalf of their family member residing in the participating facility.

## Discussion

Despite the availability of evidence-based guidelines for implementing falls prevention interventions in residential aged care, there is moderate evidence that these interventions are not routinely incorporated into routine practice. This project aims to provide a comprehensive multifaceted strategy to try to increase uptake of evidence based guidelines in the participating facilities. In line with the research evidence, a multi-factorial, individualised (to the specific requirements of each facility) and all of facility approach is planned to develop and implement an action plan based on the best available evidence in the participating facilities. We will explore the effectiveness of this approach in a range of facilities including dementia specific and psychogeriatric, in which there is limited research of effective approaches to falls prevention.

An action research approach will be used to assist in engaging staff in practice change, encouraging them to identify better practice strategies, implement and evaluate the outcomes in their own practice. It is anticipated that this approach will be useful for empowering staff and facilitating sustainable evidence based falls prevention interventions.

The collection of falls data, and an economic evaluation determining the costs of the implementation and modelling of potential cost savings through the impact of project activity will also inform whether there are economic benefits of investing in these activities. With these data, other RACFs would find it more compelling to follow a similar approach to falls minimisation.

There are numerous factors that may limit the uptake of best practice falls prevention guidelines in RACFs, including lack of knowledge among staff, residents and families that many falls are preventable and the type of interventions that can be effective; lack of optimisation of staff routines and practice; unsupportive/unsafe environments; lack of adequate falls incident reporting and analysis at the local level; and lack of staff time. This multi-factorial approach, inclusive of staff as key drivers within each facility, will implement an evidence based falls prevention project across nine Australian RACFs that aim to reduce falls and falls-related injuries.

## List of abbreviations used

RACF: Residential Aged Care Facility; FRN: Falls Resource Nurse; EFT: Equivalent Full Time; FARG: Falls Action Research Group; RPPE: Revised Professional Practice Environment Scale; FROP-RC: Falls Risk for Older People - Residential Care.

## Competing interests

The authors declare that they have no competing interests.

## Authors' contributions

BH primarily developed the paper with some sections written and the paper edited and reviewed by all remaining authors. The methodology was primarily developed by the initial chief investigators; KH, KM, TH and AR. JN and BH now have a primary role in managing all project activities in two of the three States involved in the project. All authors have read and approved the final manuscript.

## Pre-publication history

The pre-publication history for this paper can be accessed here:

http://www.biomedcentral.com/1471-2318/10/8/prepub
